# Rate of initial highly active anti-retroviral therapy regimen change and its predictors among adult HIV patients at University of Gondar Referral Hospital, Northwest Ethiopia: a retrospective follow up study

**DOI:** 10.1186/s12981-016-0095-x

**Published:** 2016-02-17

**Authors:** Degefaye Zelalem Anlay, Zinahbizu Abay Alemayehu, Berihun Assefa Dachew

**Affiliations:** Department of Nursing, College of Medicine and Health Science, University of Gondar, Gondar, Ethiopia; Department of Internal Medicine, College of Medicine and Health Science, School of Medicine, University of Gondar, Gondar, Ethiopia; Department of Epidemiology and Biostatistics, College of Medicine and Health Science, Institute of Public Health, University of Gondar, Gondar, Ethiopia

**Keywords:** Gondar, HIV infection, Rate, Regimen change, Survival analysis

## Abstract

**Background:**

Regimen change is a major challenge for the sustainability of human immunodeficiency virus (HIV) treatment program. In a resource limited setting where treatment options are limited, designing strategies to increase the durability of original regimen are essential. However, information’s on rate of initial regimen change and its predictors is scarce in Ethiopia. Therefore, the purpose of this study was to assess the rate of initial highly active anti retroviral therapy (HAART) regimen change and its predictors among adult HIV patients at the University of Gondar Referral Hospital, Northwest Ethiopia.

**Methods:**

An institutional based retrospective follow up study was conducted among 410 adult HIV patients started HAART from January 2010 to December 2014. Simple random sampling technique was used to select patient records using computer generated random number. Data were collected from patient chart using data extraction tool. The Kaplan–Meier curve was used to estimate the median duration of regimen change. Life table was used to estimate the cumulative survival for initial regimen change and log rank test to compare regimen change survival curves between the different categories of explanatory variables. Bivariate and multivariate Cox proportional hazard model were used to identify predictors of initial regimen change.

**Results:**

The overall incidence rate of initial regimen change was 10.11 (95 % CI 8.29, 12.6) per 100 person years (PY). Baseline WHO clinical stage III (AHR = 1.92, 95 % CI 1.12–3.35), occurrence of tuberculosis (TB) on the initial regimen (AHR = 8.33, 95 % CI 4.47–15.53), side effect on the initial regimen (AHR = 25.27, 95 % CI 15.12–42.00) and co-medication with ART (AHR = 2.5, 95 % CI 1.46–4.34) were significant predictors of initial regimen change.

**Conclusions:**

The rate of initial HAART regimen change was found to be high. Having WHO clinical stage III, co-medication with ART, occurrence of tuberculosis and side effect on initial regimen were independent predictors of regimen change. Hence, close follow-up and screening of patient for side effect and tuberculosis is important.

## Background

The survival of HIV patient increased due to the scale up of antiretroviral therapy and changed HIV infection into chronic conditions. However, changes in treatment and poor adherence limit the therapeutic success of original regimen and sustainability of HIV treatment program since antiretroviral therapy is a lifelong therapy. As far as there is virological success, the initial regimen continues unless the need of regimen changes due to drug side effect, co-morbid illness, pregnancy and other conditions [1–3].

Improving the long term access and sustainability of HIV treatment program by optimizing the limited available combined anti-retroviral regimen is vital. Successive regimens are inferior to that of the original regimen in related to effectiveness and duration. In addition to this; regimen change result in a number of challenges, reduce both the duration and the chance of viral control due to cross resistance between different alternative drug and overlapping toxicity between and within a class of antiretroviral (ARV) drug. Subsequently, the likelihood that successful HAART will last life time is poor [1, 2, 4, 5]. Besides, second line ART is more expensive than that of first line HAART [6].

A multi-center study in North America and Europe showed that the incidence of regimen change were 14.4/100 person years (PY) [7]. Furthermore, 13.8/100PY in Thailand [8], 41.5/100PY in Swiss [9], 28.3/100PY in Brazil [10], 16.2/100PY in West Africa [11], 18.6/100PY in Kenya [12] and the proportion of regimen change in Ethiopia was 21.8 % [13]. Different studies revealed that baseline regimen [1, 12, 14], WHO stage [12, 15], CD4 count [6, 16, 17] and patient weight [12, 15] were some predictors of initial regimen change.

In resource limited setting including Ethiopia where treatment options are limited; designing strategies to increase the durability of original regimen is essential. So as to achieve this goal, it is important to determine the rate and predictors of initial HAART regimen change. However, information’s on rate of initial HAART regimen change and its predictor in Ethiopia is scarce. Therefore, this study was aimed to determine the rate of initial HAART regimen change and its predictor among adult HIV patients. Results from this study will help to design appropriate measures to increase the duration of original regimen among patients on antiretroviral therapy (ART) which subsequently preserve the future treatment options.

## Methods

### Study design and setting

Institution based retrospective follow up study was conducted to determine the rate of initial HAART regimen change and it’s predictor among adult HIV patients at University of Gondar Referral Hospital, North West Ethiopia, from January 2010 to June 2015. The study was conducted at Gondar University Hospital ART clinic from May 25 to June 10 2015. University of Gondar Referral Hospital is a teaching Hospital which serves more than five million people of the North Gondar zone and peoples from the neighboring zones. The HIV care service of the Hospital was initiated in 2005 and has 7 outpatient rooms, one voluntary testing and counseling room, one pharmacy, and one laboratory. Since 2005 in which the hospital started ART, 7581 adults and 738 pediatrics patients are enrolled to the HIV care. Currently 4891 adults are actively following their treatment.

Standardized monitoring and evaluation tool and the data collection and management process are well organized and supported by electronic data base system of the hospital. During the study period, based on Ethiopian FMOH update for ART initiation criteria for adult and adolescent patients, the ART initiation criteria were either the CD4 count 350 cell/mm^3^ or when WHO stage III and IV disease. The choice of ART drug is based on baseline clinical (history and physical examination) and laboratory results (HGB, Liver function test, renal function test). The first line regimen consisted of NRTI backbone zidovudine (AZT) or Stavudine (d4T) or Tenofovir (TDF) or Abacavir (ABC) with Lamivudine (3TC) and either Efavirenz (EFV) or Nevirapine (NVP). Of which the preferred first line drug is TDF-3TC-EFV and the alternative first line regimen are ZDV+3TC+EFV, ZDV+3TC+NVP and TDF-3TC-EFV. After initiation of therapy the patient is appointed at 2 weeks, at 1 month, and every month for the first 6 months and then after ever 3 months. Monitoring is done through CD4 count at baseline, every 6 months but only for exceptional case viral load is done specially to assure treatment failure suspected case.

### Definition of initial regimen change

Initial regimen change defined as, a switch or substitution of at least one drug from the original HAART regimen. Regimen change, which is defined in this study, as an event, through the follow up period was ascertained retrospectively when the patients is recorded as changed their regimen and start other HAART drug. According to 2008 Ethiopian federal ministry of health ART guideline which was adopted from WHO guideline; the reasons for treatment change were based on the occurrence toxicity (grade III and IV), tuberculosis, pregnancy, treatment failure and others. Treatment failure was defined as immunological failure (fall of CD4 count to pre-Rx baseline or below or 50 % fall from the on-treatment peak value or persistent CD4 levels below 100 cells/mm^3^), clinical failure (new or recurrent WHO stage 4 condition) or virological failure (plasma VL above 10,000 c/ml for study period). Patients with the first date of lost to follow up, transfer out, death before the end of the follow up period and completed the follow up period without developed the event were considered as censored.

### Inclusion criteria

All patients aged 15 and above who were started HAART at University of Gondar Hospital, from 1st January 2010 to 31st December 2014. Within this period a total of 2521 and out of whom 410 patients record were reviewed.

### Sample size and sampling technique

The sample size was determined by using single population proportion formula through EPI INFO Stat Calc program with the assumption of 95 % level of confidence, 4 % of marginal error, and taking prevalence of regimen change 21.8 % [18]. With this consideration the final sample size became 410. Patient’s chart numbers were taken from the electronic data base as a sampling frame. From a total of 2521 patients who were started HAART 410 charts were selected and reviewed by simple random sampling technique through computer generated random number.

### Data collection tools and procedures

The available information on the patient records had been first observed and appropriate data extraction format was prepared in English. Then the data were collected by four nurses who had ART training using the prepared data collection format on the already existing records after half day theoretical and half day practical training given. One data clerk also supported them by identifying the charts. Charts were retrieved using the patient registration number which was found in the data base in the electronic system.

### Data quality control

Quality of data was maintained by recruiting data collectors who had taken ART training. The data collectors were given intensive training for one day before the data collection about the objective of the study and how to retrieve data for this study purpose using the data extraction format. They briefed on the definition of variables on the questionnaires and registration charts. The data extraction tool was pre-tested for consistency of understanding the review tools, and completeness of data items on 21 charts at the same facility as it was a secondary data and the necessary amendment were made on the final data extraction format. The retrieval process was closely monitored by the principal investigator throughout the data collection period. Completed questionnaires were checked regularly for completeness of information and any gaps identified were immediately communicated to the data collectors.

### Data processing and analysis

After the data were checked for its consistency and completeness, it was entered to EPI-INFO version 7.0 then exported to SPSS version 20 for cleaning and analysis. Summary statistics were carried out to describe the demographics, baseline and follow up data. Incidence density rate (IDR) was calculated for the entire study period. To calculate rate of regimen change among peoples on ART, the total duration of follow up for the whole cohort in person year (PY) was used. The follow up duration for peoples on ART who did not change their regimen was calculated from the time of initiation of ART until the last visit. For those who are changing their regimen; the follow up duration was calculated from initiation of HAART to a substitution of at least one drug from the original regimen. Subsequently, the number of cases who changes their regimen within the cohort was divided by the total regimen change free follow up duration and reported per 100PY.

The survival analysis technique was carried out, as this study has considered time-to-event data, Cox proportional hazard model was fitted, and a life table was used to estimate cumulative probabilities. The Kaplan–Meier curve was used to estimate the median duration of regimen change. Log rank test was used to compare survival curves between the different categories of the explanatory variables. Both bivariate and multivariate Cox proportional hazard model were used to identify the predictors. Variables with p value <0.2 in the bivariate analysis were entered into the multivariate proportional hazard model. 95 % CI of hazard ratio was computed and variable having p value <0.05 in the multivariate Cox proportional hazards model was considered as significantly and independently associated with the dependent variable. Cox proportional hazard model fitness was checked using schoenfield residual test.

### Ethical statement

Ethical clearance was obtained from the Institutional Review Board (IRB) of the University of Gondar. Permission letter was obtained from the clinical director of the Hospital. The HIV care clinic head gave the consent for extracting data from records. The confidentiality of the patient was ensured by avoiding name and identification number from extracting.

## Results

### Socio-demographic, baseline clinical and immunological status of the respondents

Four hundred ten records were analyzed. The mean age at the initiation of ART was 33.3 ± 8.7 years and 181 (44.1 %) of the participant were in the age group between 25 and 34 years. More than half of the respondents 265 (64.6 %) were female and majority 363 (88.5 %) of them were Orthodox Christian. Regarding the level of education 123 (30 %) of the respondents were completed secondary education. The majority 337 (82.2 %) of the respondents were urban dwellers. A total of 372 (90.7 %) patients had disclosed their HIV status to either their family or other relatives. Three hundred fifty nine (87.6 %) did not use any type substance (Table 1).

**Table 1:** Baseline socio demographic characteristics of HIV positive adults at initiation of HAART at University of Gondar Referral Hospital, January 2010 to December 2014 (n = 410)

Characteristics	Frequency	Percentage (%)
Age		
15–24	51	12.4
25–34	181	44.1
35–44	126	30.7
≥ 45	52	12.7
Sex		
Female	265	64.6
Male	145	35.4
Marital status		
Married	186	45.4
Divorced	96	23.4
Single	73	17.8
Widowed	31	7.6
Separated	24	5.9
Religion		
Orthodox	363	88.5
Muslim	39	9.5
Protestant	6	1.5
Catholic and Jewish	2	0.4
Level of educational		
Not educated	127	31
Primary	102	24.9
Secondary	123	30
Tertiary	58	14.1
Occupation		
Unemployed	326	79.52
Employed	84	20.48
Residence		
Urban	337	82.2
Rural	73	17.8
Disclosure status		
Disclosed	372	90.7
Not disclosed	38	9.3
Substance use		
No	359	87.6
Yes	51	12.4

The majority of the participants, 376 (91.7 %) were eligible to HAART by CD4 count of which 56 (13.6 %) were also eligible clinically. The mean weight of the Participant was 53.58 ± 9.55 kg. The predominant HAART regimen initially prescribed for them were a combination of zidovudine, Lamivudine and Nevirapine (AZT-3TC-NVP), 177 (43.2 %) followed by Tenofovir, Lamivudine and Efavirenz (TDF-3TC-EFV) for 147 (35.9 %) cases.

More than half 221 (53.9 %) of participants, were initiated on NVP based regimen. Nearly half 201 (49 %) of the patients were started by taking two ART pills/day and 401 (97.8 %) of the participant were taking CPT prophylaxis. One hundred seventy two (42 %) were WHO clinical stage III at the initiation of HAART. The majority, 361 (88 %) of the participant were on working functional status at a baseline. The median CD4 count at initiation of HAART and the end of follow up was 162.5 [IQR 90.5–235.5] and 291 [IQR 170–433] respectively. Nearly half, 192 (46.8 %) of study subjects were started HAART at HGB level of ≥ 13 g/dl. One hundred fifteen (28 %) of the study participants were diagnosed for opportunistic infection before initiation of HAART and after confirmation of HIV infection (Table 2).

**Table 2:** Baseline clinical and immunological status of HIV positive adults at initiation of HAART, University of Gondar Referral Hospital from January 2010 to December 2014 (n = 410)

Characteristics	Frequency	Percent
Initial regimen		
AZT-3TC-NVP	177	43.2
TDF-3TC-EFV	147	35.9
AZT-3TC-EFV	37	9
TDF-3TC-NVP	28	6.8
D4T(30)-3TC-NVP	16	3.9
D4T(30)-3TC-EFV	3	0.7
ABC-3TC-EFV	2	0.5
Number of ART pill/day		
One	128	31.2
Two	201	49
Three	40	9.8
Four	41	10
CPT prophylaxis		
Yes	401	97.8
No	9	2.2
Past OI before HARRT initiation		
No	295	72
Yes	115	28
Functional status		
Working	361	88
Ambulatory	29	7.1
Bed ridden	20	4.9
WHO clinical stage		
I	105	25.6
II	73	17.8
III	172	42
IV	60	14.6
CD4 count		
<100 cell/mm^3^	110	26.8
100–199 cell/mm^3^	150	36.6
200–349 cell/mm^3^	133	32.4
≥350 cell/mm^3^	17	4.1
Baseline Hgb		
<7 g/dl	8	2
7–9.9 g/dl	37	9
10–12.9 g/dl	173	42.2
≥13 g/dl	192	46.8
Base line weight		
<60 kg	318	77.6
≥60 kg	92	22.4

### Rate of regimen change

**Fig. 1:**
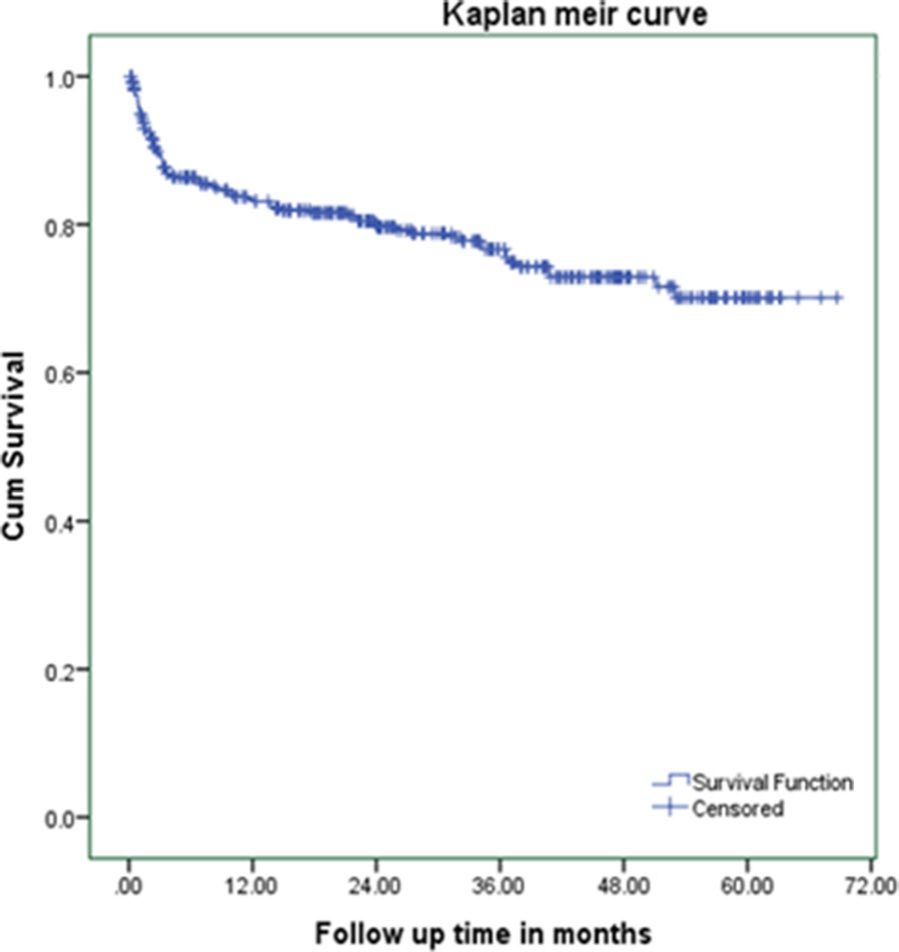
Kaplan-Meir curve of surviving on initial regimen proportion for HIV positive adults on initial HAART at University of Gondar referral Hospitals, starting ART from January 1 2010 to June 2015)

Four hundred ten study subjects who were followed for different period gave a total of 10436.2 person months (869.68 person years) of observation. Within a follow up period, a total of 88 (21.5 %) of patients were changed their initial regimen. This makes the overall rate of initial regimen change 10.11/100 PY (95 % CI 8.29, 12.6 PY). Regarding to the time of initial regimen change, 53 (60.2 %), 63 (71.6 %) and 80 (90.9 %) were changed their regimen within 6 months, 1 year and 3 years respectively, the remaining 8 (9.1 %) were changed after 3 years of follow up. The cumulative probability of surviving on initial regimen at the end of 6 months was 0.83; at the end of 1 years 0.81; at the end of 3 years 0.73 and the end of follow up was 0.7 (Fig. 1). Among the reasons for regimen change, side effect was the commonest reason which accounts for 62 (70.45 %) of cases and contribute for the 7.13/100PY. Tuberculosis 18 (20.45 %), pregnancy 4 (4.5 %), virological failure 3 (3.4 %) and occurrence of hepatitis B with chronic liver diseases 1 (1.14 %) were other reasons for regimen change. The commonest side effect for regimen change was found to be anemia 33 (53.2 %), followed by rash 14 (22.6 %) (Table 3).

**Table 3:** Common side effects among patients with regimen change at University of Gondar Referral Hospital from January 2010 to June 2015 (n = 88)

Types of side effect	Frequency	Percent
Anemia	33	53.22
Rash	14	22.58
Peripheral neuropathy	9	14.51
Nausea and vomiting	5	8.06
Abdominal pain	4	6.45
Depression	3	4.83
Lipoatropy	2	3.22
Fatigue	2	3.22

### Predictor of regimen change

**Fig. 2:**
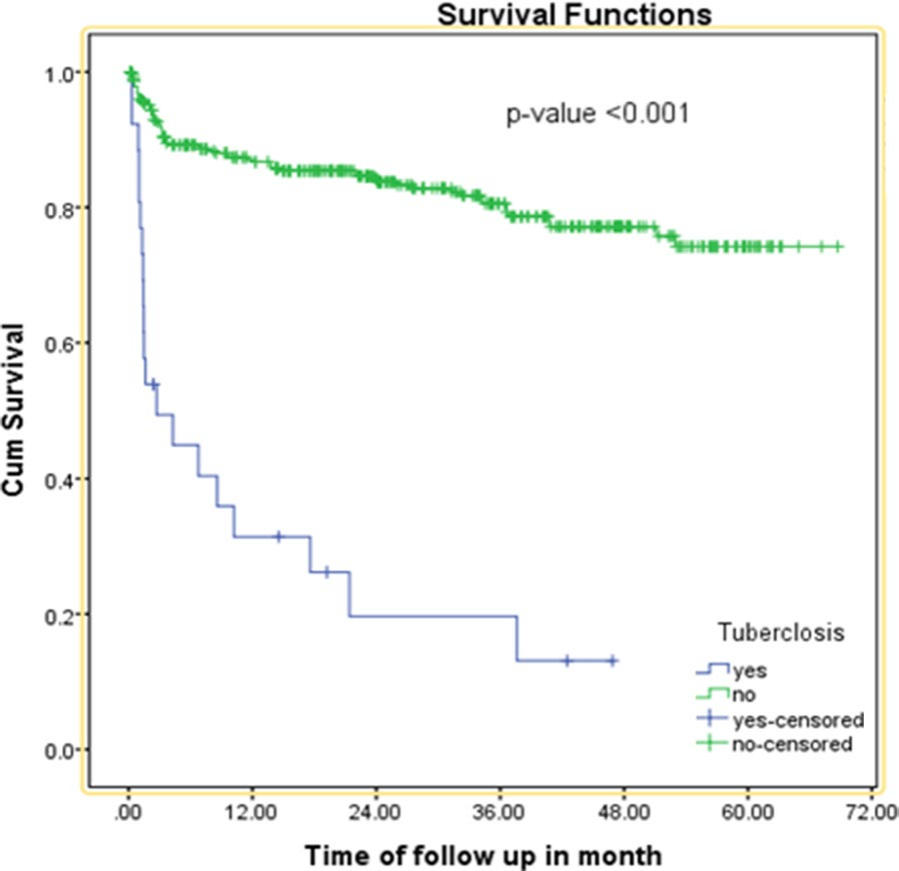
Kaplan-Meier curve of surviving on initial regimen proportion based on the occurrence of tuberculosis on initial regimen in University of Gondar Referral Hospital from January 2010 to June 2015

In the multivariate Cox-regression analysis, baseline WHO clinical stage, co-medication with ART, occurrence of TB and side effect on initial regimen remained a significant predictor of the initial regimen change. Accordingly, HIV patients who were stage III at initiation of HAART increased the risk of initial regimen change by 1.92 times when compared to those who were stage I/II at any time (AHR = 1.92, 95 % CI 1.12,3.35). Those who were developed TB on an initial regimen had about 8.33 times at higher risk of changing their initial regimen at any time as compared to those who did not develop TB (AHR = 8.33, 95 % CI 4.47, 15.53) (Fig. 2).

Patients on HAART who were taking other medication with ART had about 2.54 times at higher risk of changing their initial regimen at any time as compared to those who did not take other medication (AHR = 2.5, 95 % CI 1.46, 4.39). Those who had ART drug side effect on initial regimen were 25.27 times at higher risk of changing their initial regimen as compared to those who had not side effect (AHR = 25.27, 95 % CI 15.12–42.00) (Table 4).

**Table 4:** Bivariate and multivariable Cox regression analysis for predictors of initial HAART regimen change among adult HIV positive patients at University of Gondar Referral Hospital, January 2010 to June, 2015 (n = 410)

Variable	Survival status		Crude HR (95 % CI)	Adjusted HR (95 % CI)
	Event	Censored		
Weight				
<60 kg	56	262	1.00	1.00
≥60 kg	32	60	1.91 (1.24–2.95)	1.19 (0.70–2.03)
Baseline WHO stage				
Stage I/II	23	155	1.00	1.00
Stage III	50	122	2.45 (1.49–4.01)	1.92 (1.12–3.31)*
Stage IV	15	43	1.945 (1.01–3.73)	0.55 (0.25–1.18)
TB on initial regimen				
Yes	20	6	7.92 (4.76–13.16)	8.33 (4.47–15.53)*
No	68	316	1.00	1.00
Initial NRTI backbone				
TDF/ABC	26	151	1.00	1.00
AZT/D4T	62	171	1.68 (1.06–2.65)	072 (0.40–1.28)
Initial NNRTI				
NVP	60	161	1.84 (1.17–2.88)	1.74 (0.79–3.84)
EFV	28	161	1.00	1.00
Co-medication with ART other than CPT				
Yes	32	52	2.68 (1.74–4.15)	2.54 (1.46–4.39)*
No	56	270	1.00	1.00
Side effect				
Yes	63	9	18.93 (11.82–30.29)	25.27 (15.12–42)*
No	25	313	1.00	1.00

* Statistically significant at p value <0.05

## Discussion

The rate of initial regimen change among adult HIV patients on HAART was found to be 10.11/100PY (95 % CI 8.29, 12.6 PY). This finding is lower than a study conducted in Thailand 13.8/100PY [8], multicenter study in North America and Europe 14.4/100PY [7], Brazil 28.3/100PY [10] and Swiss 41.5/100PY [9]. This might be explained by the difference in defining outcome variables, since in our case we did not considered treatment discontinuation as regimen change unless they restart with different regimen. Furthermore, limited combined antiretroviral options or WHO based guideline in our setting may limit the clinician decision on cART modification. The other possible reasons might be regular monitoring of viral load for treatment response in developed countries might pick virological failure earlier which calls the need for regimen change.

Similarly, it is lower than studies done in West Africa and Kenya with a rate of 16.2/100PY and 18.6/100PY respectively [11, 12]. This might be due to the difference in follow up period 15 and 10.7 months in West Africa and Kenyan study but 23.28 months for our study. In addition to this our study include participants who started HAART after 2010 in which WHO recommended to phase out D4T but West Africa and Kenyan studies were done before 2011 which might overestimate the rate.

In this study, being in WHO clinical stage III at initiation of ART, occurrence of TB on initial regimen, co-medication with ART and side effect on initial regimen were found to be predictors of regimen change.

Those who had started HAART at baseline WHO clinical stage III were nearly two times at higher risk of changing their initial regimen as compared to those with WHO clinical stage I/II. This finding is in line with studies done in Switzerland and two Kenyan studies [12, 15, 16]. This might be due to the fact that those patients who had advanced disease are likely to be on other medications which might result in drug interaction, side effect which in turns result in drug change. In contrary with other most studies, in this study WHO clinical stage IV were not a predictors for initial regimen change. The possible reason might be, low survival of patient on stage four which may result in death before the occurrence of the event.

Those who had co-medication with HAART were nearly two and half times increase the hazard of changing their initial regimen at any time as compared to those who did not take other medication. This is supported by Swiss HIV cohort and Ethiopian study at Mekelle hospital [9, 18]. The possible reason might be drug—drug interaction and overlapping toxicity between ART drugs and other medication. The other possible explanation might be poly pharmacy which could lead to poor adherence due to pill burden which in turn resulted in poor efficacy of treatment result in drug change secondary to treatment failure.

Those who had side effect on the initial regimen were nearly twenty five times increased the hazard of changing their initial regimen at any given time as compared to those who did not develop side effect. This finding is supported by studies done in India, Nigeria and Ethiopia [14, 18, 19]. This is due to the fact that the occurrence of side effect alter the quality of life and even result in a death of a patient and it is a short term predictor for regimen change specially for grade III and IV toxicity but WHO recommend to follow the side effect cautiously for grade I and II toxicity before regimen change [5].

Those who had developed TB on initial regimen were nearly eight times at higher risk of changing their initial regimen at any time as compared to those who did not develop TB. This study is supported with a study done in India [19] and another literatures put it as one of the major reasons for regimen change [2, 12, 15]. In fact, tuberculosis is one of the opportunistic infection that occurs at any CD4 count in HIV patient, it might predicts clinical failure if it occurs after 6 months of initiation of ART which subsequently indicate the need of treatment change [11]. The other reason is the enzyme inducer nature of TB drugs specially Rifampicin induces cytochrome 450 enzyme that facilitate the metabolic activity of liver which makes under therapeutic level of ART drugs specially NVP which result in viral resistance, this calls for the need of regimen change when TB develop [5].The other explanation might be the pill burden result in poor adherence and its common toxicity with ART drugs result in the need for change [20].

Because of the retrospective nature of the study some important predictors which had a significant association with initial regimen change in other studies, like BMI and hepatitis B and C infection were missed.

## Conclusions

Rate of initial regimen change was found to be high and most of the change occurred within a year after initiation of HAART. Having WHO clinical stage III at initiation of ART, occurrence of TB on initial regimen, co-medication with ART and side effect up on initial regimen were found to be predictors of regimen change. Therefore, it is important to give special attention for patients who have advanced disease and taking additional medication. Strengthening care full follows up and screening for side effect and tuberculosis is important.

## Abbreviations

ABC: abacavir
AHR: adjusted hazard ratio
AIDS: acquired immunodeficiency syndrome
AZT: azidothyamidine
Cart: combined antiretroviral therapy
CHR: crude hazard ratio
CI: confidence interval
D4T: stavudine
EFV: efavirenz
HAART: highly active anti-retroviral therapy
HGB: hemoglobin
HIV: human immunodeficiency virus
IQR: interquartile range
NVP: nevirapine
OI: opportunistic infection
PLWHIV: people living with HIV
PY: persons years
SPSS: statistical package for social science
TB: tuberculosis
TDF: tenofovir
UNAIDS: Joint United Nation Program on HIV/AIDS
WHO: World Health Organization
3TC: lamivudine
